# Features of intermediate and late dry age-related macular degeneration on adaptive optics ophthalmoscopy: Pinnacle Study Report 8

**DOI:** 10.1038/s41433-025-03607-6

**Published:** 2025-01-20

**Authors:** Ahmed M. Hagag, Christopher Holmes, Ahmer Raza, Sophie Riedl, Philipp Anders, Rebecca Kaye, Toby Prevost, Lars G. Fritsche, Daniel Rueckert, Hrvoje Bogunović, Hendrick P. N. Scholl, Ursula Schmidt-Erfurth, Andrew J. Lotery, Sobha Sivaprasad

**Affiliations:** 1 Moorfields Eye Hospital NHS Foundation Trust, London, UK; 2 University College London Institute of Ophthalmology, London, UK; 3 Medical University of Vienna, Vienna, Austria; 4 Department of Ophthalmology, University of Basel, Basel, Switzerland; 5 Institute of Molecular and Clinical Ophthalmology Basel, Basel, Switzerland; 6 Ophthalmology Unit, Centro Hospitalar e Universitário de Coimbra, Coimbra, Portugal; 7 Association for Innovation and Biomedical Research on Light and Image, Coimbra, Portugal; 8 Faculty of Medicine, University of Southampton, Southampton, UK; 9 King’s College London, London, UK; 10 University of Michigan, Ann Arbor, MI USA; 11 Imperial College London, London, UK; 12 Klinikum rechts der Isar, Technical University of Munich, Munich, Germany

**Keywords:** Macular degeneration, Retinal diseases

## Abstract

**Background/Objectives:**

Adaptive optics ophthalmoscopy (AOO) has the potential to provide insights into AMD pathology and to assess the risk of progression. We aim to utilise AOO to describe detailed features of intermediate AMD and to characterise microscopic changes during atrophy development.

**Subjects/Methods:**

Patients with intermediate AMD were recruited into PINNACLE, a prospective observational cohort study. Participants underwent flood-illumination AOO using the commercially available rtx1 camera. AOO images were qualitatively assessed and correlated with clinical imaging including optical coherence tomography (OCT) and infrared scanning laser ophthalmoscopy.

**Results:**

The visibility of cone mosaic was generally compromised in eyes with intermediate AMD. We observed an association between the visibility of cone mosaic on AOO and the detection of a well-defined normal interdigitation zone on OCT. Drusen subtypes were identified on AOO as variations in reflectance at the edge and/or the centre of the druse. The absence of a hyperreflective margin was associated with the loss of the overlying ellipsoid zone on OCT prior to the collapse of the druse. With progressive attenuation of the retinal pigment epithelium and loss of photoreceptor layers, the drusenoid lesion appeared more hyperreflective with very distinctive edges.

**Conclusions:**

This cross-sectional study supports the potential value of AOO for providing information on intermediate AMD and the development of atrophic lesions that cannot be seen in conventional imaging modalities. The ongoing longitudinal PINNACLE study is assessing the significance of the described findings.

## Introduction

Age-related macular degeneration (AMD) is typically classified on colour fundus photographs according to the Beckman Initiative for Macular Research Classification.^1^ In the initial stages, eyes are stratified into early and intermediate AMD based on drusen size and retinal pigment epithelial (RPE) changes at the macula. The size of a large druse is at least 125 µm whereas the small and medium ones are smaller. Some of the eyes with intermediate AMD progress to late AMD that includes geographic atrophy (GA) and/or macular neovascularization.^1^

Drusen are not phenotyped further within this classification, but advances in optical coherence tomography (OCT) imaging have enabled the identification of subtypes of drusen including hyporeflective, cuticular, and calcified drusen, which have been linked to increased risk of progression.^2–4^ In addition, another entity termed subretinal drusenoid deposits (SDD) or reticular pseudodrusen located internal to the RPE has been identified as an independent risk factor for disease progression.^5,6^ Additional OCT markers of disease progression to GA encompass the presence of incomplete RPE and outer retinal atrophy associated with choroidal hypertransmission measuring under 250 µm (iRORA).^7^ This lesion can advance to GA, which is defined as complete atrophy of these layers accompanied by choroidal hypertransmission of 250 µm or greater (cRORA).^8^ Focal iRORA typically represents the first visible signs of atrophy. This may occur de novo or be preceded by drusen regression.

Adaptive optics ophthalmoscopy (AOO) is a relatively recent development in retinal imaging that has enabled the in-vivo visualisation of retinal cellular structures at a previously unattainable definition. AOO utilises wavefront measurement to compensate for optical aberrations using a deformable mirror or computed algorithms. The principles can be applied to light-based imaging and are mostly used in ophthalmology to augment imaging in fundus cameras, scanning laser ophthalmoscopes, and OCT.^9^ This article will focus on the use of *en face* AOO imaging with flood-illumination in eyes with non-neovascular AMD. There are limited reports describing AOO features in AMD.^10^ Studying AOO characteristics of AMD may provide insights into the pathology of the disease and prove useful in assessing the risk of progression. This article aims to describe the features seen in intermediate AMD with AOO and correlate the findings with OCT.

## Subjects and methods

The PINNACLE study is a four-country prospective observational study on prognostic modelling of patients with intermediate AMD in one or both eyes.^11^ The study received approval from the East Midlands - Leicester Central Research Ethics Committee (ref. 19/EM/0163) and adhered to the principles of Good Clinical Practice in accordance with the declaration of Helsinki.

### Patient selection

Recruited patients received verbal and written information on experimental procedures and provided written consent. The study included patients with intermediate AMD defined based on Beckman classification;^1^ eyes with large drusen (≥125 μm), or eyes with pigmentary abnormalities associated with at last medium drusen (≥63–<125 μm). Diagnosis was made by the clinical site investigator and confirmed by a central reading centre (Vienna Reading Center). Selected patients were between 55 and 90 years old with clear ocular media and pupillary dilation adequate for imaging and functional tests. Exclusion criteria included co-existent ocular disease affecting visual function or retinal morphology, glaucoma, myopia >−6D, presence of cRORA or neovascular AMD. Details on eligibility criteria were presented in the published PINNACLE protocol article.^11^ This report provides descriptive features that were observed in selected AOO images from a subset of included AMD patients in the PINNACLE study.

### Image acquisition

For the main PINNACLE study, full clinical imaging and visual function testing were performed for all patients at baseline, 12 and 24 months, including infrared reflectance scanning laser ophthalmoscopy (IR-SLO), blue autofluorescence, OCT angiography (OCTA), spectral-domain OCT (Spectralis HRA + OCT; Heidelberg Engineering, Heidelberg, Germany), AOO (rtx1 AO Retinal Camera, Imagine Eyes, Orsay, France), and microperimetry (MP, MAIA, CenterVue SpA, Padova, Italy). In addition, the OCT and IR imaging were also performed at 4 monthly intervals. Focal progression was assessed on OCT scans and was defined as drusen regression or new nascent GA.^11,12^ After identification of a focal event, targeted assessment at the focal event was performed with further OCT, OCTA, MP and AOO at 2, and 10 weeks. Performing an initial assessment within two weeks was aimed at promptly gathering detailed information about the event as soon as it was initially detected. The 10-week timepoint allows for a sufficient window of time (8 weeks from the initial assessment) during which clinically detectable changes could potentially manifest. The collection of this longitudinal part of the PINNACLE study is still in progress and is out of the scope of this report.

The acquisition protocol for OCT included high-density 20° × 20° macular scans with 193 B-scans per volume. Each B-scan was constructed from 512 A-scans and averaged from approximately 16 Automatic Real-Time (ART) frames. A simultaneous 30° IR image was captured. Macular 30° fundus autofluorescence (FAF) images were obtained with approximately 20 ART images averaged.

Standard AOO assessment included 5 horizontally overlapping images. Each image had a field of view of approximately 4° with a 2° overlap between adjacent images. Merging the 5 images would cover a macula-centred area of 12° horizontally by 4° vertically. Targeted focal event images were guided by and chosen to include local landmarks such as retinal vasculature to cover the region of interest with at least 2 overlapping images.

### Image processing and interpretation

Overlapping AOO images were merged semi-automatically using the montage function on the i2K Retina AO software (DualAlign LLC, New York, USA) which is installed standardly on the computer of the rtx1 machine. Manual identification of shared structures in adjacent images was useful for the successful execution of the montage function. The findings presented in this report represent observations made from a cross-sectional qualitative assessment of AOO images. The correlation of AOO images with clinical imaging, including OCT, IR and FAF, was performed manually with local landmarks such as retinal vessels and drusen margins used as a guide.

## Results

Although around 200 patients were imaged using AOO in the PINNACLE study, this report provides descriptive features that were observed in selected AOO images from a subset of included AMD patients.

### AOO characteristics of drusen subtypes

**Fig. 1:**
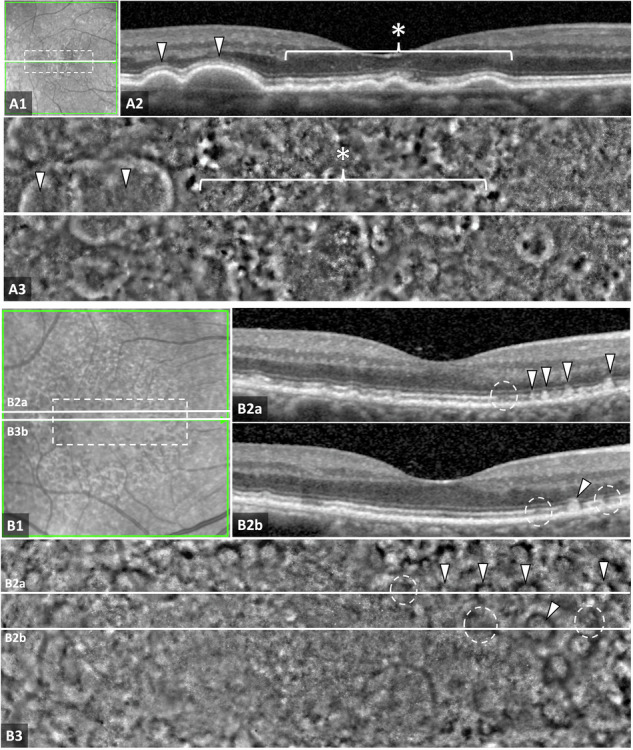
Features of drusen subtypes on adaptive optics ophthalmoscopy (AOO). The dashed box in **A1**–**B1** scanning laser ophthalmoscopy (SLO) images marks the area covered by AOO images in **A3**–**B3**. White lines in **A1**–**B1** match OCT scans in **A2**–**B2** and on AOO montage images in **A3–B3**. Large drusen look isoreflective with a clear border on AOO (**A3**, white arrowheads), surrounded by a halo of less reflectivity. Small drusen (**A2** and **A3**, asterisk) have mixed reflectivity on AOO. Subretinal drusenoid deposits (SDD) are seen in **B1-B3**. Early SDD stages (dashed circles in **B2** and **B3**) show vague hyporeflective areas on AOO (**B3**). Later stages with disrupted ellipsoid zone on OCT (**B2a** and **B2b**, white arrowheads) show a distinct ring of hyporeflectivity with a central isoreflective area (**B3**, white arrowheads).

On *en face,* AOO images, large drusen (≥125 µm) were surrounded by a nearly continuous ring of hyperreflectivity (Fig. 1, A panels). The width of the hyperreflective rim was constant regardless of the size of the large druse. This rim had no clear correlation with anatomical structures on OCT. An additional, mostly discontinuous, hyporeflective halo was observed to surround the hyperreflective rim in some cases. The centre of these drusen was generally isoreflective, i.e., it had a similar reflectivity profile to the surrounding unaffected retina.

In contrast, small and medium drusen showed heterogeneous reflectivity in the centre with areas of hypo-, hyper-, and/or isoreflectivity (Fig. 1A panels). However, not all smaller drusen possessed the hyperreflective rim with or without the discontinuous hyporeflective halo. Their variably refractile appearance on OCT did not appear to correspond with changes in AOO features.

Different stages of SDD development were observed on AOO images, with the features of the SDD becoming clearer in later stages (Fig. 1B panels). Stage 1 SDD appeared as a poorly defined hyporeflective area, with stage 2 lesions taking on the appearance of hyporeflective rings that were often incomplete, with isoreflective centres. Well-developed stage 3 SDD appeared as a well-defined hyporeflective ring with isoreflective centre, which roughly matches the characteristic doughnut shape appearance of SDD on IR images (Fig. 1B panels). Overall, AOO images showed SDD in greater numbers and with greater clarity than OCT, IR or FAF.

### Cone mosaic

**Fig. 2:**
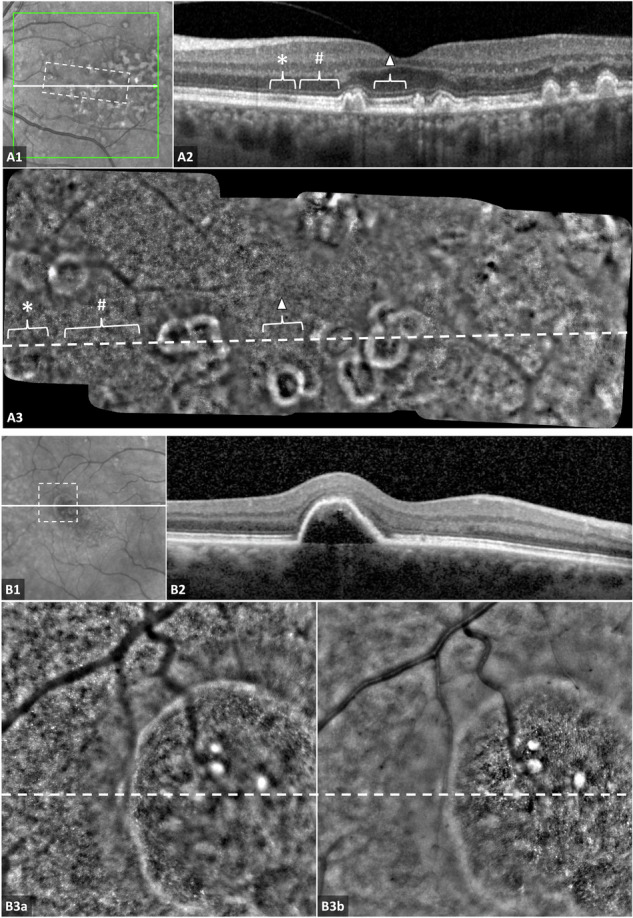
Cone photoreceptor mosaic visibility on adaptive optics ophthalmoscopy (AOO) in patients with intermediate age-related macular degeneration (AMD). Images in (**A**) and (**B**) represent two different patients. The white line in the infrared scanning laser ophthalmoscopy (SLO) images (**A1** and **B1**) correspond to cross-sectional optical coherence tomography (OCT) scans (**A2** and **B2**) and the white dashed lines in the AOO images (**A3** and **B3**a,b). The areas covered by AOO images (**A3** and **B3**a,b) are represented by the white dashed box on SLO images (**A1** and **B1**, respectively). **A** The visibility of cone mosaic on AOO is associated with the visibility of the hyperreflective band of the interdigitation zone (IZ) on OCT. Visible IZ (#, **A2**) associated with visible cone mosaic (#, **A3**). In contrast, disrupted IZ on OCT (*, **A2**) correlated with the absence of cone mosaic on AOO (*, **A3**). At the centre of the fovea, cone photoreceptors are densely packed and are too small to be resolved using this AO camera, irrespective of the status of IZ on OCT at this location (white triangle, **A2** and **A3**). **B** Exemplary images demonstrate the impact of focusing on the visibility of cones on AOO in the case of an elevated lesion (pigment epithelium detachment, PED). When focusing on the level of the normal flat retina, a cone mosaic is visible around the area of the PED, but no cones can be seen at the top of the PED (**B3**a). In contrast, when setting the focal plane on a higher level, cones become visible above the PED and disappear around the lesion (**B3**b).

The visibility of cone mosaic on AOO in AMD eyes varied due to a number of factors. At the centre of the fovea, the density of cones is too high and their sizes are too small to be resolved using this camera (Fig. 2A panels). Outside the central fovea, a strong association was observed between cone visibility and the presence of a normal intact interdigitation zone (IZ) on OCT scans. On OCT, disruption of IZ was defined by focal loss of the hyperreflective band between the ellipsoid zone (EZ) and RPE in eyes where the IZ was otherwise visible. IZ disruption on OCT corresponded spatially with poor cone visibility on AOO, even in areas with no EZ disruption or other apparent lesions on OCT scans (Fig. 2A panels). Loss of definition was also intermittently seen on and around drusen and other outer retinal lesions. Cone visibility can also vary with the change in the focal plane during image acquisition (Fig. 2B panels).

### Features of drusen regression and atrophy

**Fig. 3:**
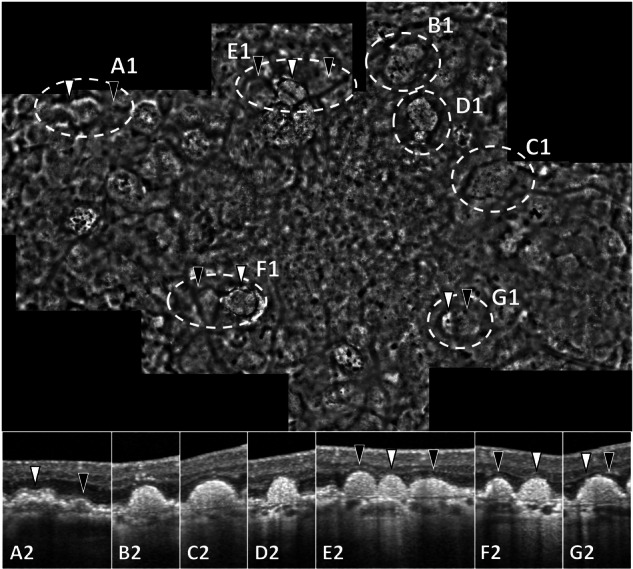
Features of drusen regression and atrophy development on adaptive optics ophthalmoscopy (AOO) in the right eye of a 67-year-old AMD patient. The top image is a montage of AOO images covering an area of up to 20 degrees horizontally and 15 degrees vertically. Optical coherence tomography (OCT, **A2**–**G2**) images at the bottom correspond to the lesions surrounded by the dashed ovals on the AOO image (**A1**–**G1**). **A** Drusen lesions with hyperreflective margin on AOO (**A1**, white arrowhead) correspond to the drusen with a relatively preserved ellipsoid zone (EZ) on OCT (**A2**, white arrowhead). The loss of EZ on OCT (**A2**, black arrowhead) is associated with the loss of the hyperreflective margin on AOO (**A1**, black arrowhead). Similarly, in (**B** and **C**), drusen lesions with disrupted EZ on OCT (**B2** and **C2**) lack the hyperreflective margin on AOO (**B1** and **C1**). Additionally, due to the retinal pigment epithelium (RPE) disruption, drusen in (**B1** and **C1**) appear slightly hyperreflective. **D** A hyperreflective lesion with a well-defined hyporeflective margin on AOO (**D1**) associated with drusen with complete loss of RPE and photoreceptor layers (outer nuclear and outer plexiform layers) on OCT (**D2**). Similar AOO lesions can be seen in (**E1** and **F1**) (white arrowheads) which also correspond to drusen with similar atrophic features. In contrast, the surrounding lesions in (**E1** and **F1**) (black arrowheads), are not hyperreflective and they lack the sharp hyporeflective edge. On OCT (**E2** and **F2**, black arrowheads), these lesions showed more preserved RPE and photoreceptor layers and less choroidal hypertransmission. **G** The left part of this lesion appears significantly more hyperreflective (**G1**, white arrowhead) than the right part (**G1**, black arrowhead). These features also corresponded to a higher degree of RPE disruption and hypertransmission in the left part of the drusen in (**G2**). Generally, early atrophic changes were more pronounced on AOO as compared to the corresponding changes on OCT.

Analysis of correlated OCT and AOO images allowed for the microscopic assessment of AOO structural changes as drusen regressed and developed into atrophy. Large drusen with minimal signs of RPE atrophy generally attained an isoreflective centre as described in an earlier section. The absence of the surrounding hyperreflectivity was often associated with the disruption of EZ on OCT (Fig. 3A). With the thinning and progressive interruption of overlying RPE on OCT, drusen appeared increasingly hyperreflective on AOO (Fig. 3B, C). Drusenoid lesions occasionally appeared hyperreflective and very well-defined with a sharply demarcated hyporeflective border on AOO, which was associated with the total loss of overlying RPE and photoreceptor layers on OCT (Fig. 3D–G). Not all collapsed drusen progressed to iRORA or cRORA. Hyporeflective clumps (HRCs) were a consistent feature at the location of regressing drusen. Hyperreflective Bruch’s membrane deposits seen on OCT in areas of atrophy appeared extremely hyperreflective on AOO, to the point that it caused all other image details to be overshadowed by the brightness compensation applied during image processing. These deposits only became visible in areas of severe RPE thinning or loss.

The correlation between OCT and AOO characteristics is summarised in Table 1.

**Table 1:** Summary of AMD features on clinical imaging and adaptive optics ophthalmoscopy.

Features on OCT	Description on AOO
Drusen subtypes (Fig. 1)	
Large drusen (≥125 µm)	External hyperreflective ring with isoreflective centre. Some drusen were additionally surrounded by a hyporeflective halo.
Small and medium drusen (<125 µm)	Areas of heterogeneous reflectivity
Subretinal drusenoid deposits (SDD)	Stage 1: poorly defined hyporeflective area Stage 2: incomplete hyporeflective ring with isoreflective centre Stage 3: well-defined hyporeflective ring with isoreflective centre SDD is better detected on AOO
Photoreceptor visibility (Fig. 2)	
Disruption of interdigitation zone	Loss of cone mosaic visibility on AOO
Drusen regression (Fig. 3)	
Disruption of ellipsoid zone on drusen	Loss of the surrounding hyperreflective ring
Thinning and interruption of overlying RPE	Centre of drusen area becomes hyperreflective
Total loss of RPE and photoreceptor layers	Drusen appear as a well-defined hyperreflective area with sharply demarcated hyporeflective border

*AMD* age-related macular degeneration, *AOO* adaptive optics ophthalmoscopy, *RPE* retinal pigment epithelium.

## Discussion

This study provides detailed AOO descriptions of drusen subtypes, cone mosaic features and different pathways of drusen regression and development of atrophy in eyes with intermediate AMD. Integrating AOO findings with conventional multimodal imaging can enhance the characterisation of established features of intermediate AMD.

In AOO, the more the light reflected from a structure reaches the detector, the more hyperreflective it would appear, and vice versa. Although the rtx1 AO Retinal Camera utilises infrared light (850 nm) in its imaging, close to that used in the Heidelberg SLO (815 nm), there are features consistently seen with AOO that were not apparent with SLO.

Firstly, we observed hyper- and hyporeflective changes surrounding large drusen. The causes of reflectivity variations at the border of drusen are unclear. Hyperreflectivity did not seem to correlate with any anatomical features on OCT. The presence of hyperreflectivity at the border of drusen is surprising as one might expect hyporeflection due to the misdirection of photoreceptors and the optical Stiles-Crawford effect (oSCE).^13^ An alternative explanation is that the hyperrelfective rim might be related to the RPE reflectance that is no longer buffered by photoreceptor outer segments. The reflectivity and contrast of drusen were previously reported to vary with gaze direction,^14^ but directional imaging did not seem to play a significant role in the variability of drusen border reflectivity. Thus, Rossi et al. proposed that the oSCE might not fully explain the mechanism of image formation and that the properties of the illumination beam need to be considered.^14^ The mitochondria in the ellipsoid layer of photoreceptor inner segments might also play a role in drusen border reflectivity. Mitochondria are thought to be a major source of near-infrared light reflectivity in the outer retina,^15^ and are likely to be the source of reflection from photoreceptors in AOO images. The relatively higher vertical density of the photoreceptor inner segments on the sloping margins of the drusen could cause a hyperreflective signal on the *en face* two-dimensional AOO images. This is supported by our observation of the association between EZ loss on OCT and the loss of drusen hyperreflective border on AOO prior to drusen collapse. Still, this hypothesis does not explain the relatively uniform size of the hyperreflective rim around drusen, irrespective of their size.

Secondly, the hyporeflective halo appeared to correspond to the change in the angle of the photoreceptors and RPE at the base of a druse, or in the trough at the confluence of 2 or more drusen. Further explorations of the light-tissue interactions are necessary to understand the complex AOO features of drusen.

Thirdly, we found that it was often difficult to discern the individual features of cuticular drusen due to their high density and indistinct margins. They had a very characteristic continuous pattern that can be clearly distinguished on AOO images. These findings are consistent with the previously reported description of cuticular drusen by other groups.^10^ The picture suggests the accumulation of widespread amorphous material under the RPE in this condition, in contrast to the visible starry sky appearance on fluorescein angiography and the closely packed tiny drusen on OCT.

Fourth, hyporeflectivity was the hallmark of SDD appearance on AOO images. The hyporeflective circle in stage 1 and the rings in stages 2 and 3 SDDs were suspected to be caused by decreased reflectance from disrupted photoreceptors at the edge of SDD development, as previously reported using AO-SLO.^16^ However, the reflectivity of the subretinal deposits varied between our flood-illumination AO system and the AO-SLO system that was used by Zhang et al. The deposits adopted a similar degree of reflectivity as the surrounding retina on FI-AO, whereas on AO-SLO, the deposits showed hyperreflectivity. This difference in reflectivity between FI and SLO highlight the need for further investigations.

Fifth, we found that the visibility of cone mosaic is reduced among the majority of intermediate AMD patients, regardless of the location of drusen/drusenoid deposits, as compared to healthy retinas. The photoreceptor signal in AO systems is largely thought to originate from the ability of cone's outer segment to waveguide reflected light.^17^ In AMD, the presence of sub-RPE drusen can cause distortion and misalignment of overlying cone outer segments, resulting in loss of their waveguiding properties. Similar to previous studies, the definition of central foveal cones was lost due to their small size and high density, although foveal cones have been visualised with other AO prototype cameras.^18^

Additionally, we observed a link between the loss of cone mosaic visibility and areas of interdigitation zone defects on OCT, in keeping with previous findings in other pathologies.^19–21^ These changes were noted even in areas of EZ preservation. The interdigitation zone represents the contact cylinder where the apical processes of RPE cells encase the photoreceptor outer segments.^22^ Previously, it was thought to display the tips of photoreceptor outer segments.^23^ Focal disruption of photoreceptor outer segments would therefore be seen as the loss of cone mosaic on AOO with corresponding change affecting the IZ more than other outer retinal bands on OCT. The EZ on OCT is thought to originate from cone inner segments and they may not contribute directly to their visibility on AOO. Verifying that EZ disruption is independently associated with loss of cone visibility will be challenging as it is unlikely to occur without the involvement of the IZ. It is also important to note that the appearance of the IZ on OCT is highly variable even in healthy eyes,^24^ and so the absence of IZ on OCT may not always imply poor cone visibility on AOO. Given all study eyes in our population had AMD, further work is required to assess whether cone mosaic loss in AOO corresponds with only diseased retina or with normal variants in retinal structure. The focusing plane is another aspect that needs to be taken into account when studying cone photoreceptors in AMD, particularly with longitudinal investigations in cases with elevated lesions, e.g., large drusen and pigment epithelial detachments.

Lastly, we were able to characterise distinct AOO morphological changes of drusenoid lesions that precede and/or accompany their collapse and atrophy formation. As the drusen grows, abnormal RPE fails to maintain the integrity of photoreceptors, which can manifest as early loss of EZ on OCT. The association between EZ disruption and the loss of the hyperreflective edge on AOO might indicate that photoreceptor inner segments contribute to this hyperreflective signal surrounding large drusen. As the RPE on top of drusen weakens, hypertransmission becomes evident on OCT, which corresponds to increased lesion reflectivity on AOO images. This hyperreflectivity is likely due to the unmasking of hyperreflective materials under the RPE. With complete loss of overlying photoreceptor layers (outer nuclear and outer plexiform layers), drusenoid lesions become well-demarcated with hyporeflective and relatively sharp edges. Decreased light scatter from dying retinal cells/layers might account for the relative clarity of the signal from drusen prior to their collapse. With the death of RPE cells, the secretion of druse components ceases, allowing for the resorption process to catch up (drusen collapse on OCT).^25^

Hyporeflective clumps were another prominent feature that commonly co-localised with collapsing drusen. The presence of HRCs might potentially signify an area of pathological activity and disease progression. HRCs were previously described in eyes with GA.^10,26,27^ It remains unclear what the HRCs represent, with proposed candidates including melanosome-containing macrophages, or free RPE cells.^10^ Further research is needed to investigate HRCs’ characteristics and their role in the pathophysiology of AMD progression.

In conclusion, this study has provided novel insights into the microscopic changes in intermediate AMD and early stages of atrophy development. The pronounced changes in the AOO reflectivity profile at the perimeter and the inside of drusen lesions might allow for early identification of areas at high risk of atrophy development. AOO features might offer useful biomarkers for prognostic modelling of AMD patients. Future research is also needed to investigate the value of AOO biomarkers in patient stratification and/or defining endpoints for clinical trials. Nevertheless, our study had several limitations. Our observations were subjective in nature and were derived from a selected subset of AOO images. Additionally, our findings were mostly based on cross-sectional observations due to the lack of large-scale longitudinal data. AOO images are also inherently limited by their relatively small field of view compared to other clinical imaging modalities. Larger studies with quantitative longitudinal assessment of AOO images are needed to objectively characterise microscopic changes in AMD. The mechanistic theory that explains the link between each AOO imaging characteristic and mitochondrial changes illustrated by changes in reflectivity of outer retinal layers and consequent inflammation and photoreceptor cell death also needs to be substantiated.

## Summary

### What was known before:

- Adaptive optics ophthalmoscopy (AOO) is able to provide microscopic features of the retina.

### What this study adds:

- The findings of this study suggest that AOO might be able to provide novel insights into the pathology and risk of progression of AMD.

## Data Availability

The datasets are available via the Vienna Reading Center, where the data will be stored, via an application to the PINNACLE consortium who are custodians of the data.
