# Correction: Features of intermediate and late dry age-related macular degeneration on adaptive optics ophthalmoscopy: Pinnacle Study Report 8

**DOI:** 10.1038/s41433-025-03696-3

**Published:** 2025-02-18

**Authors:** Ahmed M. Hagag, Christopher Holmes, Ahmer Raza, Sophie Riedl, Philipp Anders, Rebecca Kaye, Toby Prevost, Lars G. Fritsche, Daniel Rueckert, Hrvoje Bogunović, Hendrick P. N. Scholl, Ursula Schmidt-Erfurth, Andrew J. Lotery, Sobha Sivaprasad

**Affiliations:** 1 Moorfields Eye Hospital NHS Foundation Trust, London, UK; 2 University College London Institute of Ophthalmology, London, UK; 3 Medical University of Vienna, Vienna, Austria; 4 Department of Ophthalmology, University of Basel, Basel, Switzerland; 5 Institute of Molecular and Clinical Ophthalmology Basel, Basel, Switzerland; 6 Ophthalmology Unit, Centro Hospitalar e Universitário de Coimbra, Coimbra, Portugal; 7 Association for Innovation and Biomedical Research on Light and Image, Coimbra, Portugal; 8 Faculty of Medicine, University of Southampton, Southampton, UK; 9 King’s College London, London, UK; 10 University of Michigan, Ann Arbor, MI USA; 11 Imperial College London, London, UK; 12 Klinikum rechts der Isar, Technical University of Munich, Munich, Germany

**Keywords:** Macular degeneration, Retinal diseases

Correction to: *Eye* 10.1038/s41433-025-03607-6, published online 20 Jan 2025

The article “Features of intermediate and late dry age-related macular degeneration on adaptive optics ophthalmoscopy: Pinnacle Study Report 8”, written by Ahmed M. Hagag, Christopher Holmes, Ahmer Raza et al. was originally published under exclusive license to “The Author(s), under exclusive licence to The Royal College of Ophthalmologists 2025”. As a result of the subsequent decision to publish the article under the open access model, the article’s copyright notice was changed on 28. January 2025 to © The Author(s), 2025 and the article is now distributed under a Creative Commons Attribution 4.0 International License, which permits use, sharing, adaptation, distribution and reproduction in any medium or format, as long as you give appropriate credit to the original author(s) and the source, provide a link to the Creative Commons licence, and indicate if changes were made.

The images or other third party material in this article are included in the article’s Creative Commons licence, unless indicated otherwise in a credit line to the material. If material is not included in the article’s Creative Commons licence and your intended use is not permitted by statutory regulation or exceeds the permitted use, you will need to obtain permission directly from the copyright holder.

To view a copy of this licence, visit http://creativecommons.org/licenses/by/4.0.

The original article has been corrected.

